# Leaching and solvent extraction purification of zinc from Mehdiabad complex oxide ore

**DOI:** 10.1038/s41598-021-81141-7

**Published:** 2021-01-15

**Authors:** Faraz Soltani, Hossna Darabi, Reza Aram, Mahdi Ghadiri

**Affiliations:** 1 Mining Engineering Department, Arak University of Technology, Arak, Iran; 2 Mining Engineering Department, Tarbiat Modares University, Tehran, Iran; 3 Academic Center for Education, Culture and Research (ACECR) On TMU, Tehran, Iran; 4 Institute of Research and Development, Duy Tan University, Da Nang, 550000 Viet Nam; 5 The Faculty of Environment and Chemical Engineering, Duy Tan University, Da Nang, 550000 Viet Nam

**Keywords:** Chemical engineering, Mineralogy

## Abstract

An integrated hydrometallurgical process was used for the zinc leaching and purification from a zinc ore containing 9.75 wt% zinc. The zinc minerals in the ore were hemimorphite, willemite, and calcophanite. Main gangue minerals were quartz, goethite, hematite, and calcite. Central composite design (CCD) method was used to design leaching experiments and the optimum conditions were found as follows: 30% of solid fraction, 22.05% sulphuric acid concentration, and the leaching temperature of 45 °C. The PLS containing 35.07 g/L zinc, 3.16 g/L iron, and 4.58 g/L manganese impurities was produced. A special purification process including Fe precipitation and Zn solvent extraction was implemented. The results showed that after precipitation of iron, Zn extraction of 88.5% was obtained with the 2 stages extraction system composed of 30 vol% D2EHPA as extractant. The overall Zn recovery from the ore was 71.44%. Therefore, an appropriate solution containing 16.6 g/L Zn, 0.05 g/L Fe, and 0.11 g/L Mn was prepared for the electro-winning unit without using the roasting and calcination steps (conventional method), which result in environmental pollution.

## Introduction

The importance of zinc oxide ores processing such as carbonate and silicates minerals have increased because of the depletion of zinc sulphide ores and the restriction on sulphur emissions during their processing^1–6^. The zinc oxide ores usually contain several different minerals including zincite (ZnO), smithsonite (ZnCO_3_), hydrozincite (2ZnCO_3_·3Zn(OH)), hemimorphite (Zn_4_Si_2_O_7_(OH)_2_·H_2_O), and willemite (Zn_2_SiO_4_)^2^. Producing a concentrate from zinc oxide ores can be performed using flotation or gravity methods^7^. Afterwards, hydrometallurgical or pyrometallurgical routes are used for processing of the concentrate^8^. Using of pyrometallurgical routes is not suitable for processing of the low grade oxide ore due to environmental pollution, high capital investment, and significant energy consumption^9^. The main impurities associated with zinc oxide ores are calcium, magnesium, and silicon dioxide carbonates^6^. In acidic leaching, presence of soluble silicon dioxide in the oxide ore causes the formation of silica gel in the solution^6^. Different methods including using flocculent chemicals^10,11^, improvement of SiO_2_ precipitation condition^12^, SiO_2_ precipitation from colloid solutions with pH adjustment^13^, quick leaching^14^, using of microwave irradiation to quick leach^1,15^ have been successfully used and it was proved that the direct leaching methods are better than conventional roasting-leaching methods.

A number of research studies have been conducted on the alkaline leaching of zinc oxide ores. Frenay^16^ found that alkaline media is better than acidic media. The optimum alkaline leaching conditions of 5% NaOH, temperature 90 to 95 °C in 90 min was obtained for leaching zinc from an oxide ore^17^. The column leaching of low grade zinc oxide ore containing 5.2% zinc using ammonium sulphate was carried out and 92.2% of zinc was dissolved^6^. Chen et al.^8^ discovered that when the zinc oxide ores of 65–76 µm size were leached for 120 min at 358 K in the presence of 5 mol/L sodium hydroxide and liquid/solid 10:1, the leaching recovery of zinc was more than 73%. The main advantages of alkaline leaching are lower iron dissolution and lack of silica gel formation^16^. However, this method does not have the ability for dissolving of hemimorphite mineral^8^.

The Skorpion zinc processing method demonstrated the viability of the production of zinc from zinc oxide ores, using hydrometallurgical route including the leaching, solvent extraction, and electro-winning process^18,19^. However, it is difficult to separate iron from organic solution in the stripping process and SiO_2_ can be precipitated using CaCO_3_^8^. Therefore, if the solvent extraction method is used to purify the pregnant leaching solution (PLS), the iron should be removed using a suitable method before the solvent extraction stage. In hydrometallurgical processes, iron can be removed from the leaching solution in the form of hematite, goethite and various types of Jarosite^20–22^.

The obtained PLS in the leaching of oxide ores contains a number of impurities such as iron, Mn, Mg, Ca, SiO_2_, etc. Presence of iron in electrolyte can reduce zinc production and reducing current efficiency in the electro-wining unit^23^. Presence of excess levels of manganese in the electrowinning process can significantly decrease the current efficiency. Therefore, it has to be removed before entering the electrowinning process^24^. The maximum limit of Fe and Mn impurities to enter in the zinc electro-wining unit is 5 and 2000 mg/L, respectively^25^.

Solvent extraction process is a common way for selective separation or concentration of different metal ions. Purification and concentration of zinc using SX, by different organic solutions and extractants has been reported^26–29^. Extraction mechanisms of zinc using D2EHPA in an aliphatic diluent can be written as follow^30^:

1 $${Zn}^{2+}+2{(R-H)}_{org}\leftrightarrow {(Zn{R}_{2})}_{org}+2{H}^{+}$$

The (RH) is organic agent which forms organic-metal ZnR_2_ molecule using H^+^ ion. In the Eq. (1), it was assumed that the reaction takes place at organic/aqueous phases interface and water was not entered into organic phase. The zinc stripping reaction can be written as follow^18^:

2 $${(Zn{R}_{2})}_{org}+2{H}^{+}\leftrightarrow 2{(RH)}_{org}+{Zn}^{2+}$$

The present investigation is focused on zinc leaching from a zinc ore followed by iron precipitation and Zn solvent extraction and stripping. The optimum conditions for the zinc oxide leaching, solvent extraction, and stripping were determined. It should be noted that these processes alone are not novel, but the integration of these processes for the zinc oxide ores is considered as the novelty of the current research study.

## Materials and methods

### Sample preparation and characterization

A 1000 kg zinc oxide ore from the Mehdiabad mine (Yazd Province, Iran) was prepared and it was divided into two equal portions after crushing to 1-inch size using a laboratory jaw crusher and homogenization. One portion was crushed to − 2 mm particles using jaw and roller crushers. The sample was riffled to prepare representative sub-samples by using a Jones Riffler. Representative samples were milled by using a laboratory ball mill and prepared for leaching experiments. A representative sub-sample was prepared for characterization.

The elemental composition of the solution and the ore containing zinc, iron, and manganese was measured using atomic absorption spectroscopy (AAS), a Varian Spectra AA 10. Other components were analysed by x-ray fluorescence (XRF) spectroscopy technique using a PW1480 instrument (Philips Company). In the XRF method, samples were ground into a fine powder (< 75 μm), mixed with a binding aid and pressed to produce homogeneous sample pellets. Si and Al elements in the solution and ore were analysed by inductively coupled plasma-optical emission spectrometry (ICP-OES, 730-ES Varian) method. For the case of Si and Al in solid samples, alkaline fusion using lithium borate and leaching with nitric acid were performed before ICP analysis.

The ore sample were analysed by Bruker D5000 x-ray powder diffraction (XRD) system and optical mineralogy by the thin as well as polished sections. In addition, scanning electron microscope (SEM) was used to investigate smithsonite (carbonate), hemimorphite (silicate), and calcophanite (oxide) minerals.

### Zinc leaching experiments

The leaching tests were conducted in a stirred-tank reactor by H_2_SO_4_ and de-ionized water as leaching solution with agitation rate of 500 rpm, pH of less than 2 and particle size (d_80_) of 300 µm^5^. In each test, the amount of 100 g ore was added into the reactor and temperature was set at the desired temperature, then the leaching was performed for 2 h. The pH of solution was measured every 30 min. At the end of each test, the pulp was filtered, and the residue was washed a number of times by water at high temperature, then, it was dried at temperature of 105 °C. Combination of the pregnant leach solution (PLS) and wash solutions was used for the determination of zinc, iron, and manganese. All leaching samples were analysed to determine the leaching efficiency in %, as shown in Eq. (3)^31^:

3 $$R=\frac{V\times {C}_{s}}{F\times {C}_{f}}\times 100$$

where V is the PLS volume (L), C_s_ is metal concentration in the PLS (mg/L), F is the feed weight (t), and C_f_ is the grade of metal in the ore (mg/t).

### Neutralization and impurities precipitation experiments

The maximum Fe and Mn impurities concentration in the solution entering the electrowinning unit need to be 0.005 and 0.2 g/L, respectively. Therefore, it is required to conduct neutralization and impurities precipitation experiments to reduce impurities concentration in the solution. In order to conduct the iron precipitation stage, lime milk was added to the leaching pulp to increase pH value from about 2 to 4–4.5 for 2 h at 50 °C.

### Solvent extraction and stripping

Aqueous solution containing zinc, iron, and manganese for the solvent extraction process was obtained from leaching experiments. The organic phase was prepared by dissolving determined amounts of D2EHPA as extractant in kerosene as diluent. The effect of initial pH (2, 2.5, 3), vol/vol percent of extractant to diluent (10%, 20%, 30%), aqueous solution to organic (A:O) phase ratio (0.5, 1, and 2) at constant temperature, mixing time of 50 min, and agitation rate = 250 rpm were investigated. The stripping process was performed using a solution containing 220 g/L H_2_SO_4_ with the A:O phase ratio of 1 at pH 1.

## Results and discussion

### Mineralogy

#### The ore elemental composition

The ore elemental composition was given in Table 1. It can be observed that the ore contains 9.75% Zn, 20.77% Fe, 20.3% SiO_2_, 5.65% Mn, and 5.1% Al_2_O_3_.

**Table 1:** Chemical content of the studied ore.

Component	Fe	Zn	Mn	Ca	Pb	Mg	SiO_2_	Al_2_O_3_	K_2_O	L.O.I
Percentage	20.77	9.75	5.65	5.72	1.58	1.51	20.3	5.1	0.76	7.76

#### Mineralogical studies

**Figure 1:**
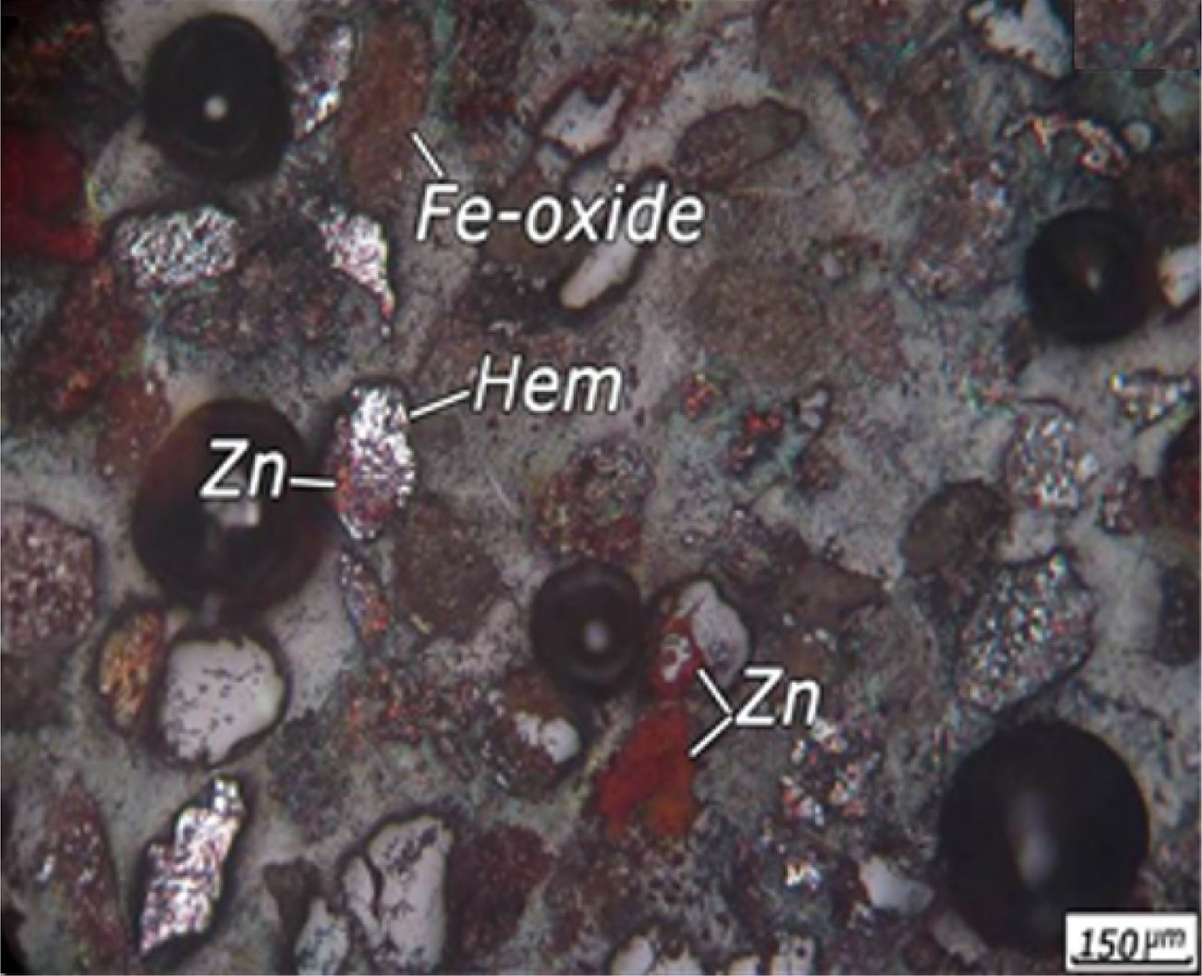
An optical microscopic image from + 90 µm size fraction showing the presence of Hem = Hematite, Zn = Zn oxide minerals, Fe-oxide = Fe oxide minerals in the ore.

**Figure 2:**
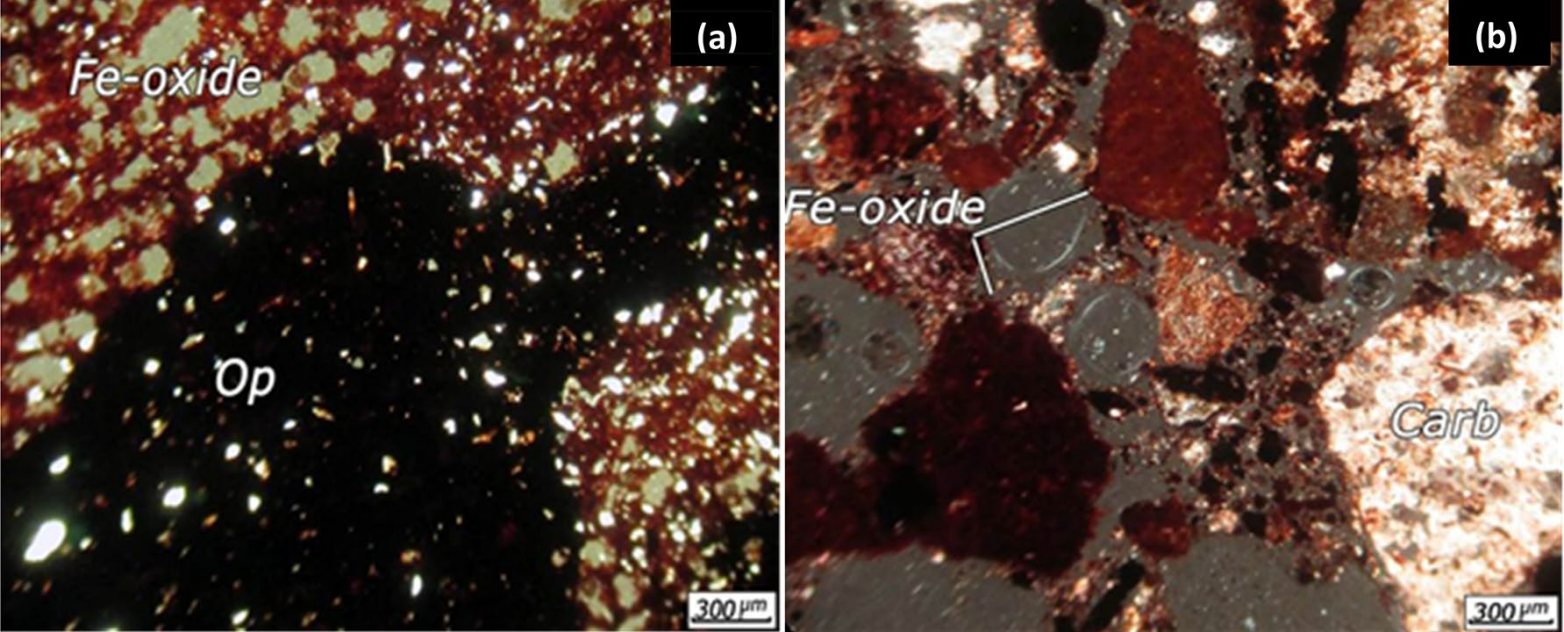
Optical microscopic images from thin section showing (**a**) association of iron minerals with carbonate minerals (**b**) free crystalline phases of iron minerals, (*Op* opaque minerals).

The XRD results showed that the dominant minerals in the ore are quartz (SiO_2_), hematite (Fe_2_O_3_), goethite (FeO(OH)), willemite (Zn_2_SiO_4_), hemimorphite (Zn_4_Si_2_O_7_(OH)_2_·H_2_O), dolomite (CaMg(CO_3_)_2_), cryptomelane (K(Na)Mn_8_O_16_), and calcite (CaCO_3_). In order to investigation of highly important minerals and their association with the gangue minerals, the polished and thin sections were studied. It was observed that metallic minerals in the polished sections are iron minerals mainly as hematite and goethite, manganese-zinc minerals mainly as calcophanite and hetaerolite (ZnMn_2_O_4_), manganese mineral as pyrplozite, and zinc minerals mainly as willemite and hemimorphite. An optical microscopic image from + 90 µm size fraction is shown in Fig. 1. It is evident from Fig. 1 that zinc minerals are mainly associated with iron oxides and manganese minerals. The willemite and hemimorphite minerals were not visible in the sections and zinc zap solution was used for the identification of these minerals. Zinc non-sulphide minerals were become red and identifiable after using the zinc zap solution. Iron minerals are usually in the form of free crystalline phases (Fig. 2b), associated with carbonate minerals (Fig. 2a) manganese oxides, and manganese-zinc oxide minerals. The iron oxide and hydroxide minerals is observed in red colour and in the form boxwork texture in Fig. 2. The iron oxides veinlets are visible between carbonate minerals (Fig. 2).

#### Minerals degree of freedom

Minerals degree of freedom in a size fraction was defined as the ratio of a valuable mineral in the form of free particles to total area of the valuable mineral in the section. Table 2 shows the degree of freedom for different size fractions. It can be observed that the zinc minerals are significantly associated with other minerals. It should be mentioned that it is not necessary to get all valuable mineral free from gangue minerals in the hydrometallurgical processes. For a valuable mineral to be leached, the contact of leaching solution with part of the mineral is enough. Therefore, leaching experiments are usually performed on different sizes to select the optimal size so that the target metal is selectively leached from other metals.

**Table 2:** Degree of freedom for different size fractions.

Size fraction (µm)	− 38	38–53	53–75	75–90	90–125	125–250	250–500	+ 500
Degree of freedom (%)	> 95	92	82	73	64	42	12	0

**Figure 3:**
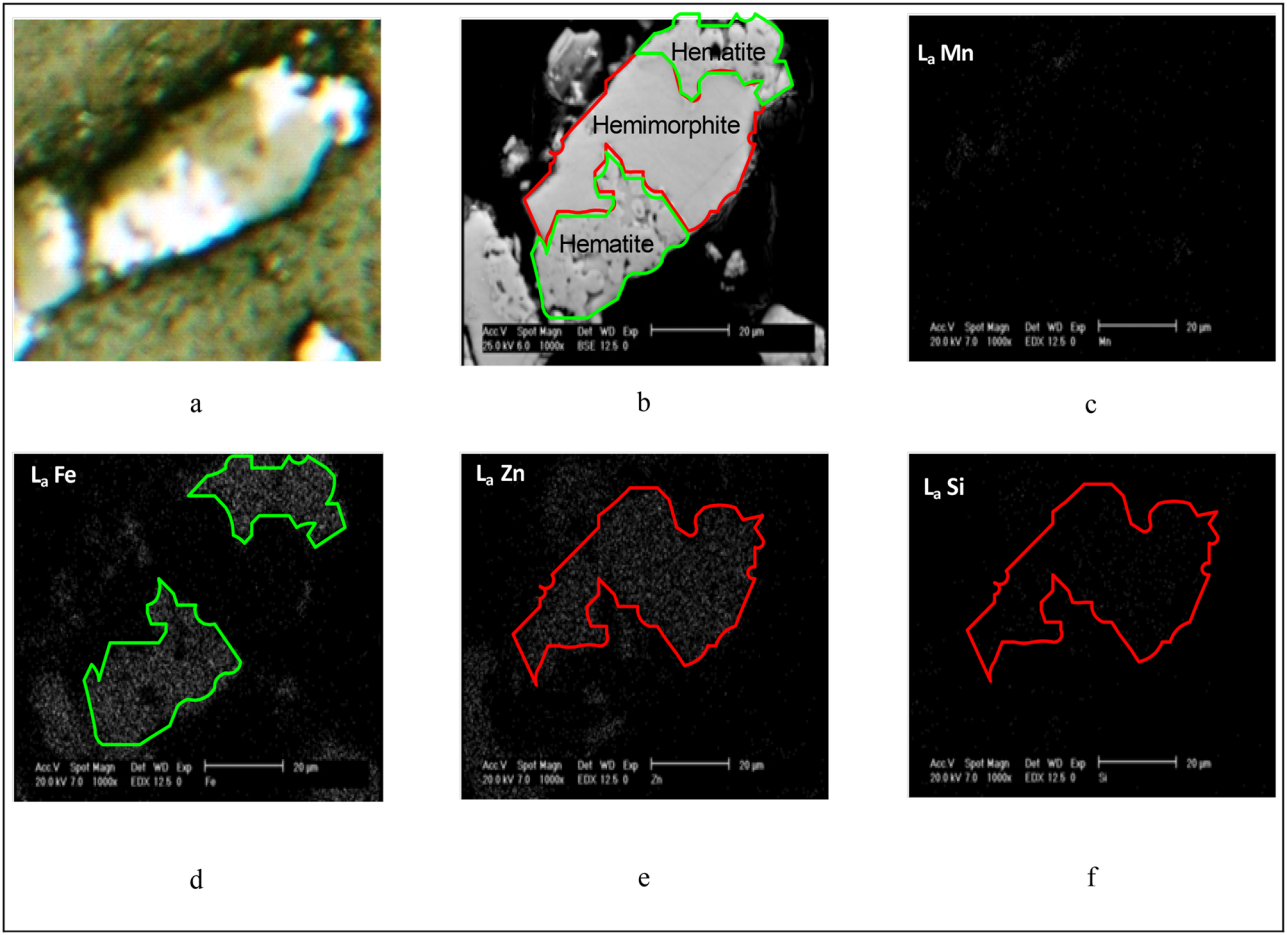
Hemimorphite association with hematite (**a**) microscopic image, (**b**) BS image by SEM, (**c**) WDX image of manganese, (**d**) WDX image of iron, (**e**) WDX image of zinc, (**f**) WDX image of Si.

The zinc minerals association with other minerals was investigated using SEM technique. The results showed that hemimorphite and smithsonite minerals are mostly close to each other. These minerals association with iron oxide minerals can be seen in Fig. 3. The calcophanite mineral was observed in different grain sizes including small and large grains. Small grains had higher association with other minerals and the grains were locked with iron minerals. However, large grains are mostly free. The large grains are divided into two groups including pure calcophanite large grains and grains containing high amount of iron.

#### Minerals composition

The ore mineral composition was approximately determined based on XRF, XRD, microscopic image, and SEM. The minerals composition of the ore is presented in Table 3. Based on the Table 3, 28.37 to 30.41%, 46.70 to 46.97%, 22.62 to 24.93% of the zinc are in the calcophanite, hemimorphite, and smithsonite minerals, respectively.

**Table 3:** The ore mineral composition.

Mineral	Amount (wt%)	Formula	Amount of Zn in the mineral (calculated %)	Zn (%)	Total Zn Distribution (%)
Dolomite	5–7	CaMg(CO_3_)_2_	–	–	
Calcite	10–12	CaCO_3_	–	–	
Smithsonite	4–5	ZnCO_3_	52.15	2.09–2.61	22.62–24.93
Goethite	12–14	FeO(OH)	–	–	
Hematite	10–12	Fe_2_O_3_	–	–	
Calcophanite	17–18	ZnMn_3_O_7_ (H_2_O)_3_	16.5	2.81–2.97	28.37–30.41
Hemimorphite	8–9	Zn_4_Si_2_O_7_(OH)_2_.H_2_O	54.29	4.34–4.89	46.7–46.97
Barite	Less than 2	BaSO_4_	–	–	
Quartz	18–19	SiO_2_	–	–	
Cerussite	Less than 2	PbCO_3_	–	–	
Psilomelane (amorphous)	8–9	(Ba,H_2_O)_2_Mn_5_O_10_	–	–	
Limonite (amorphous)	8–10	FeO(OH)	–	–	
Sum				9.24–10.47	

### Direct leaching of the ore

The solid fraction (20%, 30%, and 40%), H_2_SO_4_ concentration (12%, 18%, and 24%), and temperature (45, 60, and 75 °C) were considered as variable parameters and the particles size, leaching time, and agitation rate was kept constant in all leaching experiments. A central composite design (CCD) of experiments was carried out by Demo version of Design-Expert software version 7.0.0 (STAT-EASE Inc., Minneapolis, USA)^32^ in order to evaluate the operating parameters influence on the system performance. The preliminary experiments were conducted to find the range of changes in variable parameters.

#### The influence of solid fraction

**Figure 4:**
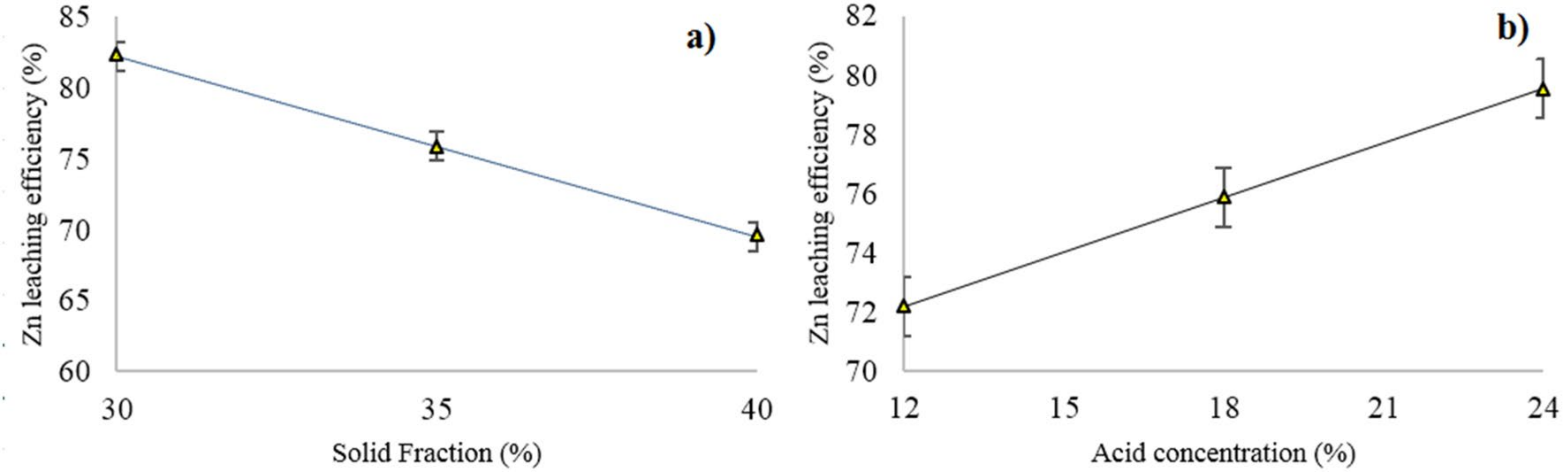
Effect of (**a**) solid fraction at H_2_SO_4_ concentration of 18% and (**b**) H_2_SO_4_ concentration at 30% solid fraction on the zinc leaching efficiency.

Effect of increasing in the solid fraction on the zinc leaching efficiency is presented in Fig. 4a. It was observed that the zinc leaching efficiency decreases from 82.22% to 69.5% with increasing the solid fraction from 30% (S/L = 3/7) to 40% (S/L = 2/3) at acid concentration of 18%. Tiechui et al.^33^ reached maximum Zn extraction of 85.69% at S/L = 1/12 (g/L) using ammonium hydroxide and ammonium chloride agents. Hua et al.^15^ investigated the quick H_2_SO_4_ leaching of a Zn silicate ore at a constant S/L = 1/10 in the presence of microwave irradiation and reached the maximum Zn extraction of 99.08%. Rao et al.^34^ studied the influence of S/L in the range of 1/10 to 1/40 g/mL and found that decreasing the ratio from 1/10 to 1/40 g/mL increased the leaching efficiency of zinc by 10%. It could be concluded that the decrease of S/L increases the mole ratio of the leaching agent to the target mineral and increases the leaching efficiency of zinc. It could be emphasized that in the aforementioned cases, high Zn leaching efficiency is obtained in the condition of small solid to liquid ratio.

#### *The influence of H*_*2*_*SO*_*4*_* concentration*

Figure 4b presents the influence of H_2_SO_4_ concentration on the zinc leaching efficiency. It was observed that the enhancement of acid concentration increased the zinc leaching efficiency. Similar results by^11^ have shown that increasing the acid concentration to 4.5 M increased the zinc leaching efficiency from hemimorphite and smithsonite up to 95%. However, it should be noted that dissolution of impurities including iron and manganese was also increased with too much increasing of acid concentration. Therefore, the acid concentration plays a key role in the leaching of zinc from oxide ores. The minimum and maximum zinc leaching efficiencies were obtained 72.18% and 79.84% at 12 wt% and 24 wt% acid concentrations (35% solid fraction), respectively.

4 $${Zn}_{4}{Si}_{2}{O}_{7}{(OH)}_{2}.{H}_{2}O+4{H}_{2}{SO}_{4}=4ZnS{O}_{4}+{{Si}_{2}O(OH)}_{6}+3{H}_{2}O$$

5 $${ZnCO}_{3}+{H}_{2}{SO}_{4}=ZnS{O}_{4}+{H}_{2}O+{CO}_{2(g)}$$

In the reactions (4) and (5), ZnSO_4_ is soluble in water solution. But, Si_2_O(OH)_6_, known as disilicic acid is polymerized to polysilicic acid and produces a gelatinous phase in acidic medium. This gelatinous phase can reduce the zinc leaching efficiency in addition to causing the filtration problem by creating an inactive layer on the zinc mineral particles^35^. As mentioned in the “Minerals composition”, hemimorphite mineral contains 46.70 to 46.97% of the total zinc in the ore. According to Table 3, if the hemimorphite mineral is completely dissolved, 1.99 to 2.24% of the total SiO_2_ in the ore enters the solution. As shown in Table 5, SiO_2_ leaching efficiency was 4.7%. Grinding the ore to d80 = 300 micron (80% finer than 300 micron) and leaching at pH around 2 could be at least part of the reason why SiO_2_ leaching efficiency is minimum. It has been reported that maintaining the pH between 1.8 and 2, maximises the stability of colloidal silica^18^.

#### Influence of temperature

The operating temperature for the zinc leaching efficiency was selected in the range of 45 to 75 °C. Preliminary results showed considerable reduction in the zinc leaching efficiency at temperatures less than 45 °C. Souza et al.^36^, showed that the leaching efficiency of hemimorphite increased with increasing of temperature from 30 to 60 °C. Abdel-Aal^37^ showed that the enhancement of the temperature from 40 to 70 °C, the Zn leaching efficiency increased from 70 to about 95%. Previous work conducted by the authors showed that increasing the temperature above 45 °C, increases the leaching efficiency of impurities^5^. Therefore, during the leaching experiments, the temperature was set at 45 °C.

#### Optimization of leaching experiments

The suggested experiment conditions by the software was shown in Table 4. The zinc and impurities concentrations in the PLS and their leaching efficiencies were given in Table 5. Maximum Zn leaching efficiency reached 83.81% at 30% solid, 22.05wt% sulphuric acid concentration, 45 °C, leaching time = 2 h as well as particle size of 300 µm (d_80_). In this condition, the leaching efficiency of Fe, Si and Al was 3.55%, 4.7%, and 4.2%, respectively. Mn leaching efficiency was 18.9% and considerable amount of Mn entered the PLS. The main reason for the low leaching efficiency of Fe, Si, Al, and Mn elements is the larger particle size of the leaching feed (d80 = 300 µm). In addition, the leaching temperature was set at 45 °C to minimize the leaching efficiency of impurities. The effect of particle size and temperature have been studied in another study by the authors^5^.

**Table 4:** Optimum conditions of the leaching experiments.

Parameter	Value	Unit
Solid fraction	30	%
H_2_SO_4_ concentration	22.05	wt%
Leaching temperature	45	°C
pH	About 2	–
Leaching time	2	H
Particles size	300	µm

**Table 5:** The zinc and impurities concentration and leaching efficiency.

Component	Leaching efficiency (%)	Concentration (g/L)
Zn	83.81	35.07
Fe	3.55	3.16
Mn	18.90	4.58
Si	4.70	1.91
Al	4.20	0.49
Sulphate ion	13.23	

### Impurities precipitation

The obtained PLS from the optimum leaching experiment requires to be prepared for electro-wining unit by removing impurities that could unselectively be extracted from the solution by D2EHPA. Therefore, firstly, the precipitation process was used to remove or decrease impurities from solution. The lime milk was added to the pulp to increase the pH of the PLS from less than 2 to about 4–4.5. Increasing the pH of the PLS could precipitate Fe(III) and metal ions as their hydroxide form. Although the precipitate produced by hydroxide precipitation method is more difficult to filter than precipitate produced in hematite, goethite and jarosite methods, but its implementation on an industrial scale has advantages such as lower temperature, less residual iron in solution (less than 0.1 g/L) and stronger ability to absorb cations and anions present in the solution.

**Figure 5:**
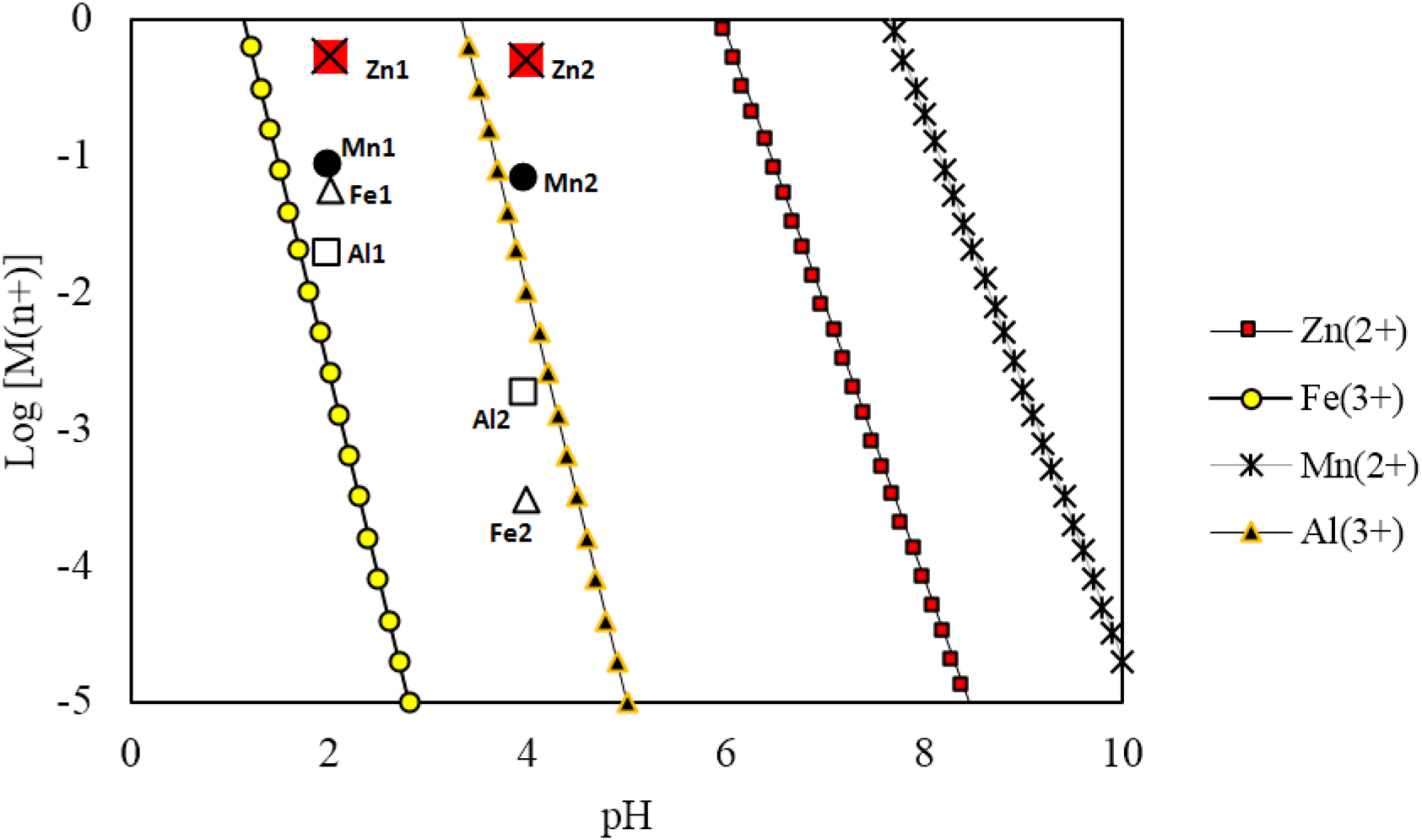
Hydroxide precipitation diagram for Fe^3+^, Al^3+^, Zn^2+^ and Mn^2+^ cations (Monhemius^38^ was used for determination of the products’ solubility). M1 means metal concentration at pH = 2 and M2 means metal concentration at pH = 4 (M = Zn, Fe, Mn, and Al).

It can be observed from Table 6 that 99.45% Fe, 18.12% Mn, 3.09% Zn, 90% Al, and 75% Si were precipitated, respectively. As shown in Fig. 5, Iron and aluminium precipitation occurs at lower pH values in comparison to Mn and Zn. Therefore, it should be pointed out that the precipitation process is remarkably effective to remove Fe impurity from the PLS while there is still significant amount of manganese in the solution. Therefore, another purification process needs to be used to remove manganese impurity from the solution.

**Table 6:** Zn, Mn, and Fe concentration before and after the neutralization stage.

Element	Precipitation (%)	Concentration (g/L)	
		Before neutralization	After neutralization
Zn	3.09	35.070	33.320
Fe	99.45	3.160	0.017
Mn	18.12	4.580	3.680
Al	90	0.490	0.048
Si	75	1.910	0.470

### Zinc solvent extraction

**Figure 6:**
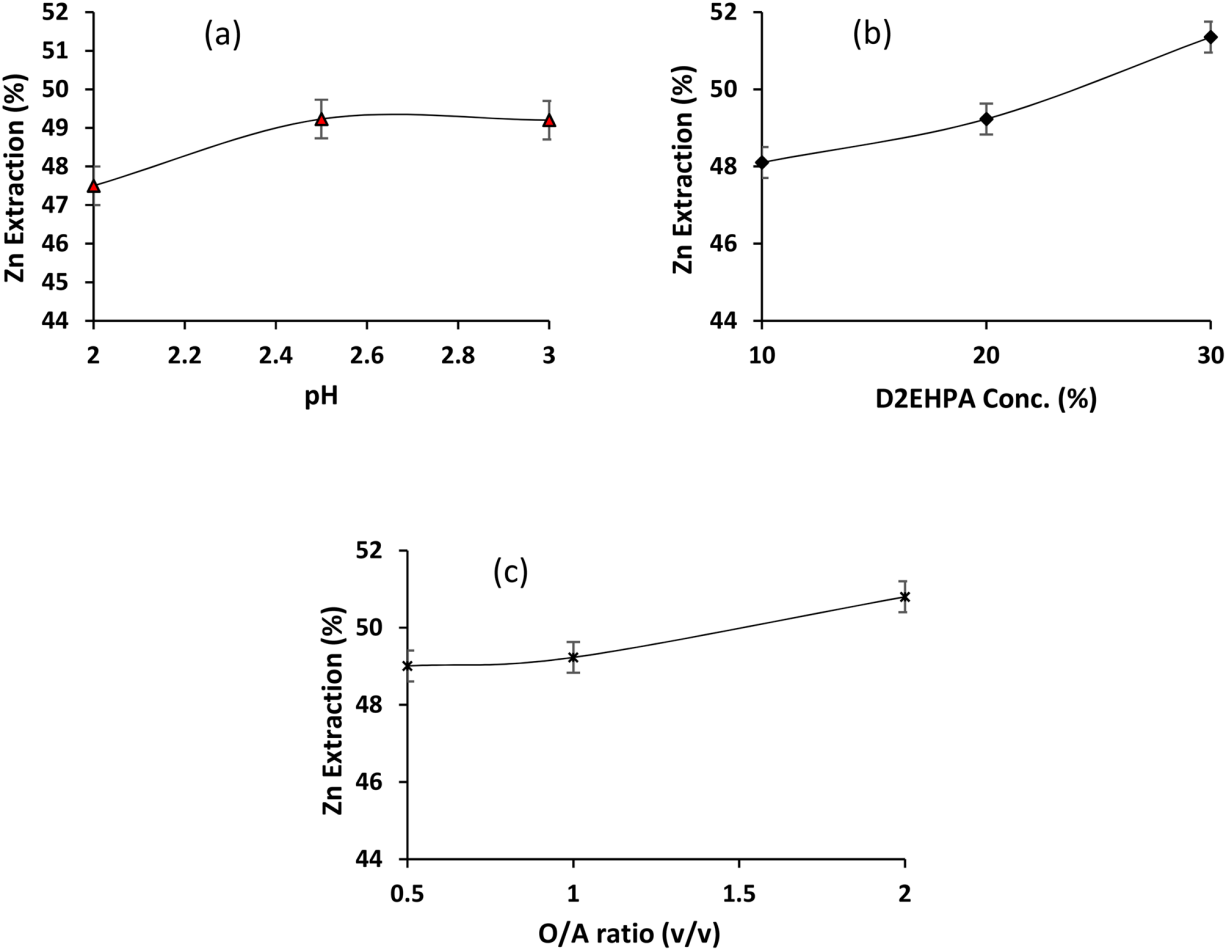
Effect of (**a**) pH, (**b**) D2EHPA concentration, and (**c**) organic phase to aqoueous phse ratio on the solvent extraction of Zn from the neutralized PLS.

A number of solvent extraction experiments at different operating conditions were designed using Design-Expert 7.0 software^32^. The influence of different operating conditions including pH (2, 2.5, and 3), O/A phase ratio (0.5, 1, and 2), and the extractant concentration (10, 20, and 30%) on the Zn extraction from aqueous solution was given in Fig. 6. By increasing the pH from 2 to 2.5, Zn extraction increased and further increasing the pH had no considerable effect on the zinc extraction (Fig. 6a). The reason for the decrease in zinc extraction at pH values below 2.5 is the protonation of D2EHPA^39^. In fact, as pH decreases, reaction 1 proceeds from right to left and H^+^ competes with zinc to react with organic matter. As shown in Fig. 6b, Zn extraction enhanced from 48.1 to 51.35% with increasing D2EHPA concentration from 10 to 30%. In addition, by increasing the organic to aqueous volume ratio from 0.5 to 2 at 20% extractant concentration, ambient temperature, and pH 2.5, zinc extraction increased from 49 to 50.8% (Fig. 6c). The maximum extraction of 52.92% was obtained under optimum operating conditions.

#### Solvent extraction experiments at optimum condition

The amount of zinc recovery was obtained 52.66% at optimum conditions. The iron and manganese recovery was found to be 66.52% and, 2.63%. As it can be seen, the iron extraction is also high. The D2EHPA extracted both zinc and iron at pH 2.5. However, this problem was solved in the stripping process because iron was remained in the organic phase.

**Figure 7:**
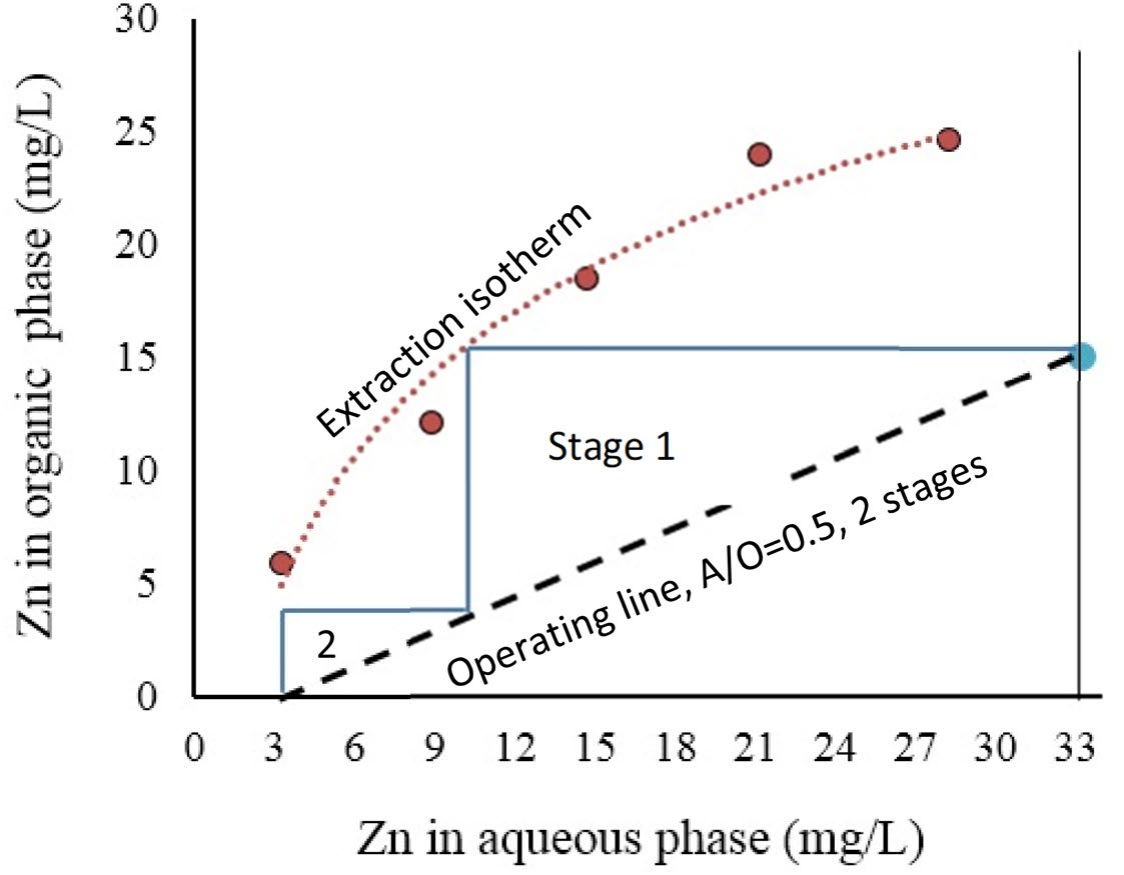
McCabe–Thiele plot of the extraction of zinc with 33% (v/v) D2EHPA. Extraction isotherm, pH = 2.5 and O:A = 0.2–5. Operating line slope (A/O) = 0.5.

The extraction isotherm was determined with combination of the neutralized solution and the organic phase at different organic phase to aqueous solution ratios from 0.2 to 5, initial pH 2.5 and 30% vol./vol. D2EHPA concentration. The extraction distribution isotherms of zinc using different O:A phase ratios is presented in Fig. 7. From Fig. 7, it was found that 2 the theoretical extraction stages are required, when organic phase to aqueous solution ratio is 2. Increasing the organic phase to aqueous solution ratio can increase the extraction concentration in the system and unnecessary enhancement of its concentration with reduce its absorption ability. Therefore, O:A ratio of 2 was selected as the best option. To verify this, A batch-continuous zinc extraction test at two stages was performed and the results are provided in Table 8. As presented in Fig. 7 and Table 7, the obtained results for the two-stage SX case show the good agreement with the predicted results using McCabe–Thiele plot. The amount of 90.91% zinc extraction was found using 30% D2EHPA concentration at two stages solvent extraction.

**Table 7:** 2 stages zinc solvent extraction.

Metal concentration (g/L)	Before SX	After SX	Recovery (%)
Zinc	33.32	3.03	90.91
Iron	0.017	0.001	94.11
Manganese	3.68	3.4	7.61

94.11% iron and 7.61% manganese were also extracted (Table 7), indicating the selectivity of the extraction process to manganese impurity. It has been reported that the order of D2EHPA selectivity is^18^:

- Fe(III) < Zinc < Calcium < Al(III) < Manganese < Cd ~ Copper < Magnesium < Cobalt < Nickel.

### Stripping of zinc

The aim of this section was to evaluate the optimum operating conditions for the stripping of zinc from the organic solution containing zinc. The zinc and impurities concentration for obtained electrolyte from the stripping process at operating conditions of A:O = 1, pH 1, and 220 g/L sulphuric acid concentration was demonstrated in Table 8. The concentration of impurities is lower than the maximum limit. Therefore, it is suitable for electro-wining unit. In addition, it was observed that the H_2_SO_4_ is an appropriate strip solution for the selective separation of zinc with 93% recovery from organic solution.

**Table 8:** Zinc, iron, and manganese concentrations in final electrolyte obtained from stripping process.

Metal	Zinc	Iron	Manganese
Concentration (g/L)	16.6	0.005	0.11

Final PLS must contain 50–60 g/L of zinc and no more than 100–150 g/L of sulphuric acid. The solution obtained in the present work after re-extraction had a zinc concentration of only 16.6 g/L (Table 8) which is not suitable for electrowinning process. Therefore, it is necessary to conduct the experiments on the extraction and re-extraction of zinc to obtain a solution with a higher concentration of zinc and a lower concentration of sulphuric acid.

## Conclusion

An integrated hydrometallurgical process for Zn leaching and solvent extraction purification from a complex zinc oxide ore was suggested. Mineralogical analyses showed that the ore contains 9.75 w.% zinc and impurities of iron (20.77 wt%) and manganese (5.65 wt%). The Maximum Zn leaching efficiency reached 83.81% at 30% solid fraction, leaching temperature of 45 °C, acid concentration of 22.05%, leaching time of 2 h, and particles size of 300 µm. One interesting result of the present work is the obtaining of pregnant leaching solution (PLS) with a high concentration of Zn (35.07 g/L) and a relatively low concentration of Fe (3.16 g/L). The PLS was purified from iron and the loss of zinc in the Fe (III) hydroxide precipitate was low (only 3%). Afterwards, 90.91% of Zn was selectively extracted over manganese by solvent extraction. An electrolyte solution with the 16.6 g/L zinc concentration was obtained in the stripping process. Presence of impurities in the solution were in the range of acceptable level for entering electro-winning unit (16.6 g/L Zn, 0.05 g/L Fe, and 0.11 g/L Mn). The overall Zn recovery from the ore was 71.44% (83.81% Zn leaching efficiency, 3.09% loss in Fe precipitation process, and 88.5% extraction-stripping efficiency). Results showed that the whole process can successfully be applied to the zinc oxide ores containing considerable amounts of Fe and Mn impurities.
